# SLEEP AND TREATMENT OUTCOME IN POSTTRAUMATIC STRESS DISORDER: RESULTS FROM AN EFFECTIVENESS STUDY

**DOI:** 10.1002/da.22420

**Published:** 2015-09-22

**Authors:** Miriam J. J. Lommen, Nick Grey, David M. Clark, Jennifer Wild, Richard Stott, Anke Ehlers

**Affiliations:** ^1^ Department of Experimental PsychologyUniversity of OxfordOxfordUK; ^2^ Oxford NIHR Cognitive Health Clinical Research FacilityOxfordUK; ^3^ National Institute for Health Research (NIHR) Mental Health Biomedical Research Centre at South London and Maudsley NHS Foundation Trust and King's College LondonLondonUK

**Keywords:** PTSD/posttraumatic stress disorder, treatment, sleep disorders, depression, CBT/cognitive behavior therapy

## Abstract

**Background:**

Most patients with posttraumatic stress disorder (PTSD) suffer from sleep problems. Concerns have been raised about possible detrimental effects of sleep problems on the efficacy of psychological treatments for PTSD. In this study, we investigated the relation of session‐to‐session changes in PTSD symptoms and sleep, and tested whether sleep problems predicted poorer short‐ and long‐term treatment outcome.

**Methods:**

Self‐reported sleep quality, sleep duration, and PTSD symptoms were assessed weekly in a consecutive sample of 246 patients who received cognitive therapy for PTSD (CT‐PTSD; Ehlers & Clark, 2000), and at follow‐up (mean = 247 days posttreatment). Additionally, moderating effects of medication use and comorbid depression were assessed.

**Results:**

Sleep and PTSD symptoms improved in parallel. The relation was moderated by depression: Sleep problems at the start of therapy did not predict improvement in PTSD symptoms during treatment for patients without comorbid depression. Patients with comorbid depression, however, showed less rapid decreases in PTSD symptoms, but comparable overall outcome, if their sleep quality was poor. Residual sleep problems at the end of treatment did not predict PTSD symptoms at follow‐up once residual PTSD symptoms were taken into account.

**Conclusions:**

CT‐PTSD leads to simultaneous improvement in sleep and PTSD symptoms. Sleep problems may reduce the speed of recovery in PTSD patients with comorbid depression. For these patients, additional treatment sessions are indicated to achieve comparable outcomes, and additional interventions targeting sleep may be beneficial. For those without comorbid depression, self‐reported sleep problems did not interfere with response to trauma‐focused psychological treatment.

## INTRODUCTION

After experiencing a traumatic event such as disaster, assault, severe accidents, or war zone experiences, many people will have problems sleeping. Among those who develop posttraumatic stress disorder (PTSD), 70–91% also have longstanding sleep disturbances, as evidenced by increased self‐reported sleep problems, and objective indicators of poorer sleep including less slow wave sleep and increased stage 1 sleep and rapid eye movement density.^1^ Sleep disturbances are seen as a core symptom of PTSD,^2^ and constitute one of the diagnostic criteria for PTSD according to the *Diagnostic and Statistical Manual of Mental Disorders*.^3^, ^4^ An emerging view in the literature, however, suggests that sleep disturbances may not just be a symptom of PTSD, but a correlated independent problem that may interfere with the efficacy of PTSD treatments and needs to be addressed directly in treatment to maximize clinical outcomes.^5^

Some experts argue that insomnia is a mechanism involved in the development and maintenance of PTSD.^5^ This view is supported by longitudinal studies showing that sleep problems before and shortly after trauma predict PTSD,^6^, ^7^, ^8^, ^9^, ^10^, ^11^ clinical studies showing that pretreatment self‐reported sleep disturbances predict lower remittance rates in primary care patients with PTSD^12^ and that treatment of insomnia leads to improvement in PTSD symptoms.^13^, ^14^

The involvement of sleep in emotional and memory processing^15^ has raised concerns about detrimental effects of sleep disturbances on cognitive behavioral therapy (CBT) efficacy.^16^ Experimental studies indicate that sleep promotes retention of fear extinction and extinction generalization,^17^, ^18^, ^19^ although not all studies have found an effect of sleep on extinction memory.^20^ Most trauma‐focused psychological PTSD treatments include some elements of exposure to trauma memories and trauma reminders. As extinction learning is seen as a mechanism of exposure therapy,^21^ an important clinical question is whether sleep disturbances impair therapeutic gains of trauma‐focused psychological PTSD treatments.

There is as yet insufficient evidence on the relation between sleep and PTSD, and the effects of sleep problems on treatment effects in people with PTSD. If sleep disturbance is a symptom of PTSD, sleep would be expected to improve concurrently with other PTSD symptoms during PTSD treatment. CBT for PTSD has indeed a moderate effect on sleep disturbance according to a meta‐analysis (ES = 0.404).^22^ Insomnia decreases in PTSD patients who show a clinical significant response to PTSD treatment,^23^, ^24^ and improvements may be maintained for years after treatment completion.^25^ However, sleep disturbance, in particular insomnia, does not seem to benefit as much as other PTSD symptoms from empirically supported PTSD treatments,^5^, ^26^ and insomnia is the most common residual symptom that typically remains in the clinical range when PTSD is targeted in treatment.^23^, ^24^

Moreover, there is a growing body of literature showing that insomnia is predictive of other psychiatric disorders including new‐onset and recurrent depression.^27^, ^28^, ^29^ Comorbid depression is common in PTSD (typically around 50%).^30^ There is a large literature linking sleep problems and depression.^29^ It is thus of clinical importance that insomnia is effectively targeted in the treatment of PTSD with and without comorbid depression.

Previous studies on the relation between changes in sleep and PTSD during therapy have been limited by focusing on pre‐ to posttreatment changes, consequently prohibiting any analyses on the course of symptoms across therapy. To investigate the nature of the relation between sleep and PTSD more closely, this study analyzed session‐to‐session changes in sleep duration and quality and PTSD symptoms in a consecutive sample of patients who received cognitive therapy for PTSD (CT‐PTSD).^31^ The aims of this study were to investigate (1) the relation between changes in PTSD symptoms and sleep across treatment, (2) whether sleep problems at the beginning of treatment predicted poorer response to treatment, and (3) whether residual sleep problems at the end of treatment predicted poorer long‐term outcome, over and above what could be predicted from PTSD symptoms at the end of treatment. Expanding on research question 2, this study also explored the effects of medication use and comorbid depression, as both might affect sleep and PTSD pathology. Findings with regard to the effect of these variables on treatment outcome in patients with PTSD have been mixed, with medication use as a moderator of treatment outcome in some studies,^32^ but not others,^33^ and comorbid depression as a moderator of treatment outcome in some studies,^[34,35]^ but only a nonspecific predictor in others.^32^, ^33^ This study explored whether medication use or a comorbid major depressive disorder at the start of treatment moderated the effects of sleep problems on PTSD symptom change. Investigation of these research questions can inform therapists whether it is necessary to specifically target sleep problems in patients with PTSD.

## METHOD

### PARTICIPANTS AND PROCEDURE

The patients eligible for this study constitute a consecutive subsample (*n* = 246) of a cohort study of 330 patients aged 17–83 who received psychological treatment for PTSD at a National Health Service outpatient clinic serving a defined catchment area in South London between April 2001 and August 2008. Weekly sleep measures were introduced from September 2003 onwards. The local research ethics committee approved the study. Details can be found elsewhere.^33^ Patients met diagnostic criteria for PTSD according to the Structured Clinical Interview for DSM‐IV (SCID).^36^ On average, the traumatic event addressed in treatment happened 37.43 months before initial assessment (range 2.50–360 months). At initial clinical interview, 91.9 % of the patients reported sleep problems, with 72.2% reporting sleep onset problems, 91.9% mid sleep awakening, and 57.5% early morning awakening. See Table 1 for demographic information.

**Table 1:** Demographic information (*N* = 246)

	*n* (valid %)
Gender	
Male	98 (40)
Marital status	
Single	103 (43)
Married or cohabiting	90 (38)
Divorced or widowed	47 (20)
Educational degree	
None	41 (17)
GCSE (school qualification taken at age 16) or equivalent professional education	99 (42)
A‐level degree (school qualification taken at age 18)	39 (17)
University level degree	56 (24)
Race	
White	138 (56)
Black	63 (26)
Multiracial	44 (18)
Trauma type sought treatment for	
Interpersonal trauma	141 (57)
Accidents	59 (24)
Event where another person was harmed or died	19 (8)
Another kind of traumatic experience	27 (11)
Comorbid Axis‐I disorder	195 (79)
Comorbid anxiety disorder	107 (44)
Comorbid depression	129 (52)
Taking psychotropic medication (stable dose for at least 2 months before treatment started)	102 (46)

The majority of the patients included in this study (*n* = 158, 64.23%) also provided data at long‐term follow‐up, which on average took place at 247 days after the last therapy session. Compared to patients who provided long‐term follow‐up data, those who did not reported more PTSD symptoms and poorer sleep quality and duration at the end of the therapy, all *P*s < .01, and were more likely to be divorced or widowed, have a lower educational degree, have a race other than white, and have been diagnosed with another Axis‐I disorder, all *P*s < .05.

### TREATMENT

Patients received a course of CT‐PTSD^37^; a trauma‐focused psychological treatment that is based on Ehlers and Clark's cognitive model of PTSD.^31^ It aims to change cognitive processes that maintain the perception of current threat in PTSD patients, by focusing on the nature of the trauma memory, problematic appraisals, and behavioral and cognitive strategies that maintain the appraisals and problematic memory features. Details of treatment procedures are found at http://oxcadat.psy.ox.ac.uk/downloads%20and%20links. The treatment does not target sleep directly. Patients received on average 10.30, *SD* = 5.16, weekly therapy sessions, and optional monthly booster sessions, *M* = 1.81, *SD* = 1.75. The analyses of session‐to‐session changes in this study focus on the first 10 therapy sessions to ensure sufficient valid data per data point.

### MEASURES

Patients were interviewed by a trained clinician with the full SCID at initial assessment and completed the following self‐report questionnaires to assess PTSD symptoms and sleep at initial assessment and before each treatment session. This ensured that for each patient, including dropouts, data at the last treatment session (including any booster sessions) were available. Measures were also collected at 6‐month and 1‐year follow‐ups. Data from the last available follow‐up were used for the analysis (*M* = 247 days after the last session).

#### PTSD Symptoms (Excluding Sleep)

PTSD symptom severity was measured with the Posttraumatic Diagnostic Scale (PDS).^38^ The PDS is a 17‐item self‐report questionnaire assessing PTSD symptoms as specified in the DSM‐IV.^3^ All items are rated on a 4‐point scale (0 = *never* to 3 = *5 times per week or more / almost always*). To avoid content overlap with the sleep measures, a revised sum score of 16 items, excluding the item “Difficulty falling or staying asleep,” was used in the analyses (PDS_wos_).

#### Sleep Measures

Sleep quality was assessed by asking patients to rate the quality of their sleep (*Overall, how well did you sleep?*) during the past week on a scale between 0 (not at all well) and 100 (very well). Sleep duration was assessed by asking patients to report the mean number of hours they slept per night in the past week (*In the past week, how much sleep did you have per night?*). The sleep duration rating correlated strongly with the sleep duration according to clinical interview at initial assessment, *r* = .83.

#### Depression

Current comorbid major depression was assessed with the SCID,^36^ referring to the last month.

### DATA ANALYSIS

Parallel process latent growth modeling (LGM) was used (Mplus version 7.11)^39^ to analyze the session‐to‐session changes in PTSD symptoms (excluding the sleep item; PDS_wos_) and sleep. Participants who provided data on PTSD symptom severity and sleep for at least three of 10 weekly sessions were included in the analyses, resulting in a sample of 207 and 211 patients to assess the relation between sleep quality and PTSD symptom severity, and the relation between sleep duration and PTSD symptom severity, respectively. Missing (sum) scores on PDS_wos_ or sleep variables in the LGM analyses were estimated using full information maximum likelihood. All analyses were conducted both for the relation between PDS_wos_ and sleep quality, and the relation between PDS_wos_ and sleep duration.

The parallel process LGM followed a bottom‐up strategy, starting with investigating variance (individual differences) between participants in PDS_wos_ or sleep variables starting level (intercept), and variance in the change in PDS_wos_ or the sleep variables across treatment (slope). For more details on the specific models (see online Appendix A). Model fit was evaluated using fit indices chi square, comparative fit index (CFI), and root‐mean‐square error of approximation (RMSEA). Chi‐square values were used to compare model fit of different models. CFI values of >0.90 and RMSEA values of <0.08 were regarded as adequate fit.^40^ Once the best model was selected, research questions 1 and 2 were investigated by testing the correlation between the slope of the sleep variable and the slope of the PDS_wos_, and testing the predictive effect of the intercept of the sleep variable on the slope of the PDS_wos_, respectively. For the latter analysis, potential moderating effects of medication and comorbid depression were also tested.

To investigate research question 3, regression analyses with the last available sleep rating and PDS_wos_ rating at the end of treatment were included as independent variables predicting the PDS_wos_ at the last available long‐term follow‐up assessment.

**Figure 1:**
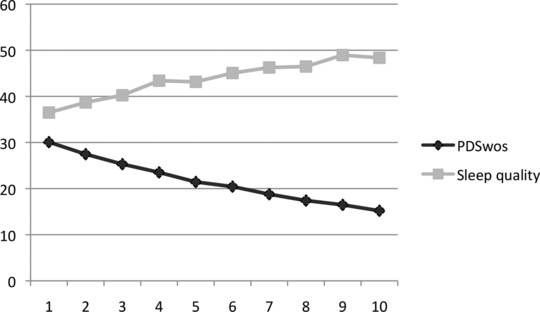
Changes in PTSD symptoms (excluding sleep; PDS_wos_) and sleep quality over 10 treatment sessions (*n* = 207).

## RESULTS

### DESCRIPTIVE STATISTICS

As shown in Table 2, PDS_wos_ scores decreased by 50% (mean change = 15.69) from initial assessment to the end of treatment in the intent‐to‐treat sample, whereas sleep quality ratings increased by a mean of 21.60 and sleep duration improved by 52 min.

**Table 2:** Descriptive statistics on PTSD symptoms and sleep variables at initial assessment, last session, and follow‐up (intent‐to‐treat)

	Initial assessment			Last session			Follow‐up		
	*M*	*SD*	*n*	*M*	*SD*	*n*	*M*	*SD*	n
PTSD symptoms without sleep item (PDS_wos_)	31.09	8.34	246	15.40	13.56	246	11.89	11.32	158
Sleep quality	33.70	21.16	231	55.30	25.28	233	55.78	29.27	148
Sleep duration (in hours)	5.14	1.70	240	6.01	1.84	238	6.16	1.62	153

### POSTTRAUMATIC SYMPTOMS AND SLEEP QUALITY

See Figure 1 for a graphic display of changes in PTSD symptoms and sleep quality over 10 treatment sessions. As shown in Table 3, the random intercept/random slope model showed best fit (model 4a). The absence of an improvement in model fit by the addition of the intercept–slope correlations within variables (model 4b) suggests that the level of PTSD symptoms at the start of treatment did not correlate with the degree of improvement in PTSD symptoms across treatment, and the starting level of sleep quality did not correlate with the degree of improvement in sleep quality. Since the addition of a quadratic term to the random intercept/random slope model (model 4a) for the PDS_wos_, *μ* = 0.08, *p* < .001, improved model fit, *∆χ*² = 16.86, *∆df* = 1, *P* < .001 (model 5a), but not for sleep quality (model 5b), model 5a was used to test research questions 1 and 2.

**Table 3:** PTSD symptoms and sleep quality (*n* = 207)

					PDS_wos_		Sleep quality		PDS_wos_		Sleep quality	
Model	*χ*²	*df*	CFI	RMSEA	*μ_i_*	*σ_i_*²	*μ_i_*	*σ_i_*²	*μ_s_*	*σ_s_*²	*μ_s_*	*σ_s_*²
1. Fixed intercept	2,210.84	217	0.32	0.21	22.64	–	42.69	–	–	–	–	–
2a. Random intercept	1,175.82	215	0.67	0.15	22.59	97.02	42.99	305.47	–	–	–	–
2b. 2a with intercept correlation^a^	1,083.87	214	0.70	0.14	22.58	97.20	42.92	306.42	–	–	–	–
3. Random intercept, fixed slope	491.26	212	0.91	0.08	29.02	103.68	37.96	312.85	−1.61	–	1.24	–
4a. Random intercept, random slope	359.79	210	0.95	0.06	29.05	87.09	37.92	273.09	−1.65	0.90	1.27	3.33
4b. 4a with intercept–slope correlation within constructs^b^	359.35	208	0.95	0.06	29.05	86.23	37.94	283.35	−1.65	0.88	1.27	3.68
5a. 4a with quadratic term for PDS	342.93	209	0.96	0.06	29.87	87.44	37.91	273.12	−2.33	0.87	1.27	3.33
5b. 4a with quadratic term for sleep quality	357.26	209	0.95	0.06	29.05	87.09	36.79	273.44	−1.65	0.90	2.12	3.29

PDS_wos_ = Posttraumatic Diagnostic Scale sum scores excluding sleep item.

a The addition of the correlation between the intercepts of PDS_wos_ and sleep quality, *r* = −0.69, *P* < .001, contributed significantly to the model fit, ∆χ² = 91.95, ∆*df* = 1, *P* < .001, and this correlation was therefore retained in models 3–5.

b The addition of the correlation between the intercept and slope within PDS_wos_, *r* = 0.02, *P* = .80, and sleep quality, *r* = −0.07, *P* = .56, did not significantly contribute to the model fit, and was therefore not retained in the next models.

To assess the relation between change in sleep problems and change in PTSD symptoms (question 1), the correlation between the slope of sleep quality and the slope of PDS_wos_ was tested. Model fit was good, *χ*² = 319.78, *df* = 208, CFI = 0.96, RMSEA = 0.05. The correlation between the slopes was significant, *r* = −0.60, *P* < .001, and improved the model fit, *∆χ*² = 5.90, *∆df* = 1, *P* = .02, showing that the decrease in PDS_wos_ correlated with the increase in sleep quality with treatment.

To assess whether initial sleep problems predicted poorer response to treatment (question 2), the effect of the intercept of sleep quality on the slope of PDS_wos_ was added to the latter model. Model fit was good, *χ*² = 318.06, *df* = 207, CFI = 0.96, RMSEA = 0.05. The intercept of sleep quality did not predict the slope of PDS_wos_, *β* = −0.13, *P* = .19 and did not improve the model fit significantly, *∆χ*² = 1.72, *∆df* = 1, *P* = .19. Further analyses used cross‐lagged path analysis to test the temporal relationship between changes in sleep quality and changes in PTSD symptoms. The results indicate simultaneous changes, and a bidirectional relationship between sleep and PDS_wos_, as sleep problems predicted changes in PTSD_wos_, and PTSD_wos_ also predicted changes in sleep problems (see online Appendix B).

### POSTTRAUMATIC SYMPTOMS AND SLEEP DURATION

The analyses including PTSD symptoms (PDS_wos_) and sleep duration showed comparable results. Based on the random intercept/random slope model (model 5a; Table 4), the slope of sleep duration correlated significantly with the slope of PDS_wos_, *r* = −0.63, *P* < .001. The intercept of sleep duration did not predict the slope of PDS_wos_, *β* = −0.09, *P* = .35.

**Table 4:** PTSD symptoms and sleep duration (*n* = 211)

					PDS_wos_		Sleep duration		PDS_wos_		Sleep duration	
Model	*χ*²	*df*	CFI	RMSEA	*μ_i_*	*σ_i_*²	*μ_i_*	*σ_i_*²	*μ_s_*	*σ_s_*²	*μ_s_*	*σ_s_*²
1. Fixed intercept	2,609.97	217	0.33	0.23	22.40	–	5.51	–	–	–	–	–
2a. Random intercept	1,215.39	215	0.72	0.15	22.27	96.06	5.50	1.95	–	–	–	–
2b. 2a with intercept correlation^a^	1,161.66	214	0.74	0.15	22.27	96.21	5.50	1.96	–	–	–	–
3. Random intercept, fixed slope	563.48	212	0.90	0.09	28.75	103.16	5.28	1.96	−1.61	–	0.06	–
4a. Random intercept, random slope	455.70	210	0.93	0.07	28.80	87.52	5.28	1.90	−1.67	0.90	0.06	0.01
4b. 4a with intercept–slope correlation within constructs^b^	455.15	208	0.93	0.08	28.81	85.21	5.28	1.90	−1.67	0.87	0.06	0.01
5a. 4a with quadratic term for PDS	437.51	209	0.94	0.07	29.65	87.88	5.28	1.90	−2.36	0.86	0.06	0.01
5b. 4a with quadratic term for sleep quality	453.42	209	0.93	0.07	28.80	87.51	5.22	1.90	−1.67	0.90	0.10	0.01

PDS_wos_ = Posttraumatic Diagnostic Scale sum scores excluding sleep item.

a The addition of the correlation between the intercepts of PDS_wos_ and sleep duration contributed significantly to the model fit, and this correlation was therefore retained in models 3–5.

b The addition of the correlation between the intercept and slope within PDS_wos_ and sleep duration, respectively, did not significantly contribute to the model fit, and was therefore not retained in the next models.

### MODERATOR ANALYSIS

To test for possible moderation effects of psychotropic medication, medication use and the interaction between the intercept of sleep quality (or duration) and medication use were added together with the intercept of sleep quality (or duration) as predictors of the slope of PDS_wos_. In the sleep quality analysis, medication use significantly predicted the slope of PDS_wos_, *P* = .02; use of medication was related to slower improvement in PDS_wos_ during treatment. The intercept of sleep quality and its interaction with medication use did not significantly predict the slope of PDS_wos_, *P =* .91, and *P* = .23, respectively. In the sleep duration analysis, neither medication use, the intercept of sleep duration, nor the interaction term predicted the slope of PDS_wos_, *P* = .13, *P* = .92, and *P* = .37, respectively.

Comorbid depression significantly moderated the effect of the intercept of sleep quality on the slope of PDS_wos_: comorbid depression and the interaction term significantly predicted the slope of PDS_wos_, *P* = .001, and *P* = .01, respectively, but the intercept of sleep quality did not, *P* = .61. A multigroup model explored this interaction effect. The intercept of sleep quality did not predict the slope of PDS_wos_ in patients without comorbid depression, *β* = 0.08, *P* = .61, but did predict the slope of PDS_wos_ in patients with comorbid depression, *β* = −0.45, *P* = .001. However, there was no moderating effect of comorbid depression in the analysis of sleep duration: comorbid depression, the intercept of sleep duration, and the interaction term did not predict the slope of PDS_wos_, *P* = .55, *P* = .52, and *P* = .97, respectively.

In sum, both sleep measures (sleep quality and duration) increased across treatment, whereas PTSD symptoms (PDS_wos_) decreased. The increase in both sleep measures correlated highly with the decrease in PDS_wos._ The intercepts of the sleep measures did not predict the slope of the PDS_wos,,_ with the exception that the intercept of sleep quality, but not sleep duration, predicted a slower decrease in PDS_wos_ for patients with comorbid depression. A regression analysis further tested whether this also applied to final treatment outcome: PDS_wos_ at the start of treatment, *β* = 0.47, *P* < .001, and comorbid depression, *β* = 0.28, *P* < .001, significantly predicted PDS_wos_ at the last session, but not sleep quality or its interaction with depression.

### LONG‐TERM TREATMENT OUTCOME

For the subsample of patients who provided data on the long‐term follow‐up (*n* = 158), the average PDS_wos_ score was 11.35, *SD* = 10.90, at the end of treatment and 11.89, *SD* = 11.32, at follow‐up, indicating stable treatment effects. Sleep quality, *M* = 59.40, *SD* = 24.62; *M =* 55.87, *SD* = 29.27, and sleep duration, *M* = 6.25, *SD* = 1.89; *M* = 6.16, *SD* = 1.62, remained similar from end of treatment to follow‐up, respectively.

Regression analyses showed that sleep quality predicted PDS_wos_ at follow‐up when considered as the sole predictor, β = −0.46, *P* < .001, *R* ^2^ = 21.3%, but was no longer a significant predictor, β = −0.08, *P* = .16, after inclusion of PDS_wos_ at the end of treatment, β = 0.74, *P* < .001, *R* ^2^ = 61.8%. Similarly, sleep duration at the end of treatment predicted PDS_wos_ at follow‐up, β = −0.40, *P* < .001, *R* ^2^ = 16.3%; but was not longer a significant predictor β = −0.09, *P* = .10, when PDS_wos_ at end of treatment was included, β = 0.75, *P* < .001; *R* ^2^ = 62.6%.

## DISCUSSION

The aim of this study was to investigate the relation between changes in PTSD symptom severity and sleep problems in a clinical sample of PTSD patients receiving trauma‐focused cognitive therapy for PTSD that did not directly target sleep. Session‐to‐session decreases in PTSD symptoms correlated highly with session‐to‐session increases in sleep quality and duration. Interestingly, sleep quality and sleep duration at the start of treatment did not predict the decrease in PTSD symptom severity across treatment. Thus, the level of sleep problems at the start of treatment was not related to poorer treatment outcome in the whole sample. However, additional analyses showed a moderating effect of comorbid depression: sleep quality at the start of treatment predicted slower change in PTSD symptoms per treatment session, but not poorer overall outcome, for patients with comorbid depression, but not for those without depression. This moderating effect of comorbid depression was not found in the analyses including sleep duration. Both self‐reported sleep quality and sleep duration at the end of treatment predicted PTSD symptom severity at follow‐up, on average 247 days later, but was no longer predictive when PTSD symptom severity at the end of treatment was taken into account, suggesting that sleep did not predict unique variance in long‐term treatment outcome.

Overall, the findings do not support the hypothesis of detrimental effects of the presence of sleep problems on the efficacy of cognitive behavioral treatments for PTSD,^16^ with the exception that for patients with comorbid depression poor sleep quality at the start of treatment predicted a slower decrease in PTSD symptoms over the first 10 sessions, but not final treatment outcome. Possibly sleep problems associated with depression are more severe or differ qualitatively from those associated with anxiety problems alone,^41^, ^42^ with only the former interfering with rapid gains in therapy. It is unclear whether this is due to biological, psychological, or social factors. Comorbid depression in patients with PTSD has been related to social problems like unstable housing conditions,^33^ which could interfere with sleep directly and could make the treatment less trauma focused, resulting in a slower treatment response. Moreover, patients with comorbid depression might have been less motivated or might have had less energy to do assignments outside of the sessions, resulting in a slower recovery. However, the moderating effect of comorbid depression was not replicated in the analyses on the effect of sleep duration on PTSD symptoms, and should therefore be interpreted with caution. Other sleep factors that have been strongly related to insomnia, such as sleep latency, wake time after sleep onset, sleep maintenance difficulties, and perceived sleep‐related daytime symptoms, might have had a stronger influence on sleep quality than on sleep duration ratings. This may help to explain the discrepancy in findings between these two sleep variables.

On average, CT‐PTSD led to an increase of nearly 1 hr in self‐reported sleep duration (see Table 2). This is a substantial improvement, given that CBT for insomnia leads to an average improvement of 30–60 min in sleep duration.^43^ The improvement in sleep duration observed with CT‐PTSD is also well within the range of sleep gains observed when sleep is directly targeted in PTSD. A study of combat veterans with PTSD found an increase of 30 min with four sessions of CBT for insomnia and imagery rehearsal therapy for nightmares (from 4.70 to 5.20 hr of sleep).^[^14^]^ Although several studies have found that sleep problems remained in the clinical range in treatment responders who received PTSD treatment,^23^, ^25^ the average sleep duration at the end of treatment in the intent‐to‐treat sample (6.01 hr) lies within one standard deviation of the national representative sample (*M* = 7.04, *SD* = 1.55),^44^ suggesting that reduced sleep duration increased to a nonclinical level.

But how does PTSD treatment affect sleep? One possibility may lie in the arousal associated with PTSD.^1^ A study on CBT for insomnia found that reduced arousal and hypervigilance related to better treatment outcomes.^45^ Since CT‐PTSD leads to improvements of arousal and hypervigilance, it may directly improve sleep too. Likewise, reducing re‐experiencing symptoms including nightmares may result in less wakening and less avoidance of sleep. For some patients sensory cues associated with going to bed (e.g., darkness, lying down) are powerful triggers of re‐experiencing symptoms, which they learn to discriminate from the traumatic situation in therapy. Furthermore, patients learn to drop coping strategies that interfere with sleep such as worry and rumination. Finally, even though sleep problems were not directly targeted in treatment, some of the procedures may have had some overlap with advice on sleep hygiene, for example, some patients worked on reducing avoidance of going to bed for fear of nightmares or intruders. It is also conceivable that the weekly self‐ratings about sleep quality and duration might have had a small therapeutic effect itself, but as these were not discussed with the therapist, it is unlikely that this would account for the large change observed in this study.

A few limitations should be taken into account. First, simple self‐report scales were used to measure the average sleep quality and duration over a week, and objective measures of sleep that may be able to detect more subtle relations between sleep and PTSD symptoms were not included. However, although sleep duration is underestimated in self‐report measures compared to objective sleep measures, these are correlated in a way that those with the shortest sleep duration according to objective measures also self‐report the shortest sleep duration.^46^ Furthermore, the measures showed convergent validity since similar results were obtained for both sleep quality and sleep duration. Second, because this study included a patient sample, it is unclear if sleep problems were already present before the trauma and may have contributed to PTSD vulnerability, or whether sleep problems developed as a response to the trauma. Investigating whether there are common risk factors underlying PTSD and sleep disturbances might be an interesting future direction. Third, the sleep problems might not have been severe or long‐lasting enough to interfere with PTSD treatment. This seems unlikely, since the study investigated a clinical sample of PTSD patients with moderate to severe chronic PTSD who experienced the traumatic event on average 3 years before the start of treatment and had not recovered since. Fourth, it is unclear whether the results can be generalized to other types of treatment for PTSD that rely more heavily on exposure. Fifth, follow‐up analyses were restricted to the 64% of the patient sample that reported follow‐up data. Because this group reported less PTSD and sleep pathology at the end of treatment than those patients who did not provide follow‐up data, it is unclear if the results generalize to patients with higher levels of symptomatology at the end of treatment.

The strengths of this study include use of a representative sample of consecutively referrals, and the combination of the longitudinal design of assessing sleep and PTSD symptoms at each treatment session with the statistical method to analyze the data, which allowed studying the course of the symptomatology as well as a sophisticated way of dealing with missing data. Furthermore, the data collection method made sure that outcome scores at the last treatment session were available for every patient, including dropouts.

This study provides a clear answer to the clinically relevant question of whether clinicians should be hesitant to start trauma‐focused psychological therapy for PTSD if the patient reports significant sleep problems: overall, self‐reported levels of sleep problems at the start of treatment did not predict treatment effectiveness. However, in those with comorbid depression, lower self‐reported sleep quality predicted slower improvement in PTSD symptoms. It therefore appears advisable to assess depression and sleep disturbances in patients with PTSD. Although these symptoms appear to be part of the PTSD syndrome and improve in parallel with PTSD symptoms during trauma‐focused treatment, patients with comorbid depression and sleep problems required more treatment sessions to achieve comparable outcomes. It remains to be tested whether they might further benefit from additional interventions targeting sleep or depression that could be added to the trauma‐focused treatment. It also remains to be tested whether treating remaining sleep problems with either specific psychological sleep interventions, or medication that have shown to be effective in reducing sleep complaints in PTSD (e.g., olanzapine, prazosin)^47^, ^48^, ^49^ could further improve sleep. In conclusion, most people with PTSD report sleep problems and only for those with comorbid depression poorer self‐reported sleep quality, not sleep duration, was related to slower recovery, but not treatment outcome at the end of treatment. For those without comorbid depression, self‐reported sleep problems do not affect the degree to which they benefit in the short or long term from trauma‐focused cognitive therapy for PTSD.

## Disclosure of conflict of interest

Drs. Ehlers, Wild, and Stott were funded by The Wellcome Trust during the duration of the study. The funder had no role in the study design; the collection, analysis, or interpretation of data; the writing of the manuscript; or the decision to submit the paper for publication. Drs. Ehlers, Clark, Grey, Wild, and Stott received honoraria for workshops on the treatment of PTSD. Dr. Lommen reports no conflicts of interest.

## APPENDIX A

To investigate variance in the intercept (starting level) and slope (change across treatment) of PDS_wos_ and the sleep variable, the goodness of fit for the following models was computed (see Tables 1 and 2).

1. A null model (fixed intercepts), which equates to the null hypothesis of no change over time or variation between subjects for both the PDS_wos_ and sleep variable.
2. A random intercept model, in which variation of the intercepts was allowed (2a). The addition of the correlation between the intercepts of the PDS_wos_ and sleep variable was also evaluated (2b).
3. A random intercept, fixed slope model, which assumed a similar change over time for all subjects.
4. A random intercept/random slope model, in which variation between subjects in intercept and slope was allowed (4a). The addition of the correlation between the intercept and slope of the PDS_wos_ and the correlation between the intercept and slope of sleep was also evaluated (4b).
5. Additionally, we tested whether adding a quadratic term to the growth curve estimation for the PDS_wos_ (5a) or for the sleep variable (5b) increased model fit.

## APPENDIX B

### CROSS‐LAGGED RELATIONSHIP PTSD SYMPTOMS AND SLEEP

A cross‐lagged panel analysis was run including autoregressive paths, correlation between the sleep variable and PTSD symptoms (PDS_wos_) at each time point, and cross‐lagged paths that were set to be equal over time. The fit of the model testing the temporal relationship between sleep quality and PDS_wos_ was at the boundary of adequate model fit, *χ*² = 424.00, *df* = 160, CFI = 0.91, RMSEA = 0.09. Cross‐lagged paths were all significant, with PDS_wos_ as a highly significant predictor of sleep quality, *B* = −0.38, *P* < .001, as well as sleep quality significantly predicting of PTSD symptoms, *B* = −0.02, *P =* .01. Omitting the cross‐lagged paths with PDS_wos_ as a predictor of sleep quality led to decreased model fit, *∆χ*² = 61.34, *∆df* = 1, *P* < .001. Omitting the cross‐lagged paths with sleep quality as a predictor of PDS_wos_ also led to decreased model fit, *∆χ*² = 6.68, *∆df* = 1, *P* = .01. Sleep quality predicted 2–4% of variance in PDS_wos_ across weekly sessions, and PDS_wos_ predicted 1–6% of the variance in sleep quality, both over and beyond the variation explained by autoregressive paths and the correlation between sleep quality and PDS_wos_. These results indicate a bidirectional relationship between PDS_wos_ and sleep quality.

The cross‐lagged path analysis including sleep duration and PDS showed a similar pattern of results, but did not reach adequate model fit.
